# NY-ESO-1- and survivin-specific T-cell responses in the peripheral blood from patients with glioma

**DOI:** 10.1007/s00262-017-2066-z

**Published:** 2017-10-20

**Authors:** Zhenjiang Liu, Thomas Poiret, Oscar Persson, Qingda Meng, Lalit Rane, Jiri Bartek, Julia Karbach, Hans-Michael Altmannsberger, Christopher Illies, Xiaohua Luo, Inti Harvey-Peredo, Elke Jäger, Ernest Dodoo, Markus Maeurer

**Affiliations:** 1 Therapeutic Immunology, F79, Department of Laboratory Medicine, Karolinska University Hospital Huddinge, Karolinska Institutet, Hälsovägen, Huddinge, 14186 Stockholm, Sweden; 2 Centre for Allogeneic Stem Cell Transplantation (CAST), Karolinska University Hospital, Stockholm, Sweden; 3 Department of Neurosurgery, Karolinska University Hospital, Stockholm, Sweden; 4 Department of Clinical Neuroscience, Section for Neurosurgery, Karolinska Institutet, Stockholm, Sweden; 5 Department of Neurosurgery, Copenhagen University Hospital Rigshopitalet, Copenhagen, Denmark; 6 Department of Oncology and Hematology, Krankenhaus Nordwest, Frankfurt/Main, Germany; 7 Institute of Pathology, Krankenhaus Nordwest, Frankfurt/Main, Germany

**Keywords:** Glioma, Survivin, NY-ESO-1, Cellular therapy, T-cells, Immunotherapy

## Abstract

The prognosis for patients with glioblastoma is grim. Ex vivo expanded tumor-associated antigen (TAA)-reactive T-cells from patients with glioma may represent a viable source for anticancer-directed cellular therapies. Immunohistochemistry was used to test the survivin (*n* = 40 samples) and NY-ESO-1 (*n* = 38 samples) protein expression in tumor specimens. T-cells from peripheral blood were stimulated with TAAs (synthetic peptides) in IL-2 and IL-7, or using a combination of IL-2, IL-15 and IL-21. CD4^+^ and CD8^+^ T-cells were tested for antigen-specific proliferation by flow cytometry, and IFN-γ production was tested by ELISA. Twenty-eight out of 38 cancer specimens exhibited NY-ESO-1 protein expression, 2/38 showed a strong universal (4+) NY-ESO-1 staining, and 9/40 cancer lesions exhibited a strong (4+) staining for survivin. We could detect antigen-specific IFN-γ responses in 25% blood samples for NY-ESO-1 and 30% for survivin. NY-ESO-1-expanded T-cells recognized naturally processed and presented epitopes. NY-ESO-1 or survivin expression in glioma represents viable targets for anticancer-directed T-cells for the biological therapy of patients with glioma.

**Electronic supplementary material:**

The online version of this article (doi:10.1007/s00262-017-2066-z) contains supplementary material, which is available to authorized users.

## Introduction

Glioblastoma multiforme (GBM) has a grim prognosis [1]. The current standard treatment modalities are limited to surgery, radiation and temozolomide [2]. New treatment modalities are needed for patients with GBM, including adjunct cellular therapies targeting tumor-associated antigens (TAAs) in order to achieve better clinical outcomes [3]. Several TAAs have already been described, such as the spliced form of the EGFR, i.e., EGFRvIII [4, 5]. This mutation induces increased migration of tumor cells and is associated with radiation and chemotherapy resistance [6]. EGFRvIII has been demonstrated to be a tumor-specific antigen (TSA) for T-cell and B-cell responses [7] in biological therapy for patients with GBM. Other TAAs include the IL-13Rα2, that is preferentially expressed in glioma, yet not in non-transformed cells [8]. IL-13Rα2 may be targeted by CD8^+^ T-cell responses or by chimeric antigen receptor-engineered T-cells (CARs) [9, 10]. The IL-4R has also been found to be overexpressed in glioma cells, and IL-4 fused to a Pseudomonas toxin [IL-4 (38–37)–PE38KDL] has been shown to induce apoptosis of IL-4R-positive glioma cells [11]. A different tumor-associated antigen (TAA) is survivin, a member of the ‘family of inhibitors of apoptosis’ which is involved in cell cycle progression and apoptosis control [12]. Survivin is overexpressed in a number of human cancers [13], associated with poor prognosis [14], and able to stimulate survivin-reactive CD8^+^ T-cells [15, 16]. NY-ESO-1, a member of the cancer–testis antigens, is expressed in about 20–40% of cancers [9, 17] and represents a promising target for anticancer-directed immune responses [17]. NY-ESO-1 was originally identified by serum screening (SEREX) and has been shown to induce potent MHC class I- and class II-restricted T-cell responses [18]. Several other shared TAAs have been identified in GBM, including members of the MAGE family, neu/c-erbB-2, gp100 or MART-1/Melan-A [19]. NY-ESO-1 expression was reported to be low in glioma (1/30 glioma testing positive for NY-ESO-1), yet demethylating agents have been shown to increase TAA expression [20] leading to improved presentation of TAAs to T-lymphocytes. We tested survivin and NY-ESO-1 expression in brain cancer sections and examined TAA-reactive cellular immune responses in the corresponding blood samples from patients with glioma.

## Materials and methods

### Diagnosis and patients

Forty-six patients (Table 1) were enrolled in the study; the study was approved by the Regional Ethical Review Board at Karolinska Institutet, Stockholm, Sweden (Dnr: 2013/576-31). Patients were divided into groups (Table 1) based on the WHO histopathological glioma grade (II, III, IV), as well as according to histology, i.e., oligoastrocytoma/oligodendroglioma (OA/O), astrocytoma (A) and glioblastoma multiforme (GBM).

**Table 1:** Summary of patient characteristics

Patient characteristics	*N* = 46
Age median (years)	58 years
Age range (years)	30–76 years
Sex (male/female)	31/15
Diagnosis	
Glioblastomas	
Grade IV	30
Astrocytomas	
Grade II	6
Grade III	3
Oligodendrogliomas	
Grade II	1
Grade III	2
Mixed (0A)	
Grade II	2
Grade III	2
Relapse tumors	3
Secondary tumors	3
Corticosteroids	Betamethasone
Median (days)	15 days
Range (months)	0–9 min
< 2 weeks	27/46
> 2 weeks	19/46

### Immunohistology

#### NY-ESO-1 staining

Tumor tissue sections were cut, deparaffinized and treated with 3% H_2_O_2_ in order to block endogenous peroxidase activity and to ‘demask’ for antigen retrieval. Forty tumor samples were available for survivin staining with sufficient amount of tumor material present for immunohistopathology. Either to the scarcity of resected tumor material available, or due to non-sufficient representative tumor material in individual sections, only 38/40 samples could be analyzed for NY-ESO-1 protein expression. The primary monoclonal mouse anti-NY-ESO-1 (clone E978) provided by the Monoclonal Antibody Core Facility (MACF) at Sloan Kettering Institute for Cancer Research [21], diluted in antibody diluent, was added for 30 min. Sections were then incubated with horseradish peroxides-conjugated ENVision for 30 min. The final reaction was visualized by incubation with diaminobenzidine + substrate–chromogen, followed by counterstaining of sections with hematoxylin. Each tissue section was semiquantitatively scored based on the intensity of immunostaining as negative (0 tumor cells stain positive), focal (less than 5% of tumor cells stain positive) or positive (score 1 = 6–25%, score 2 = 26–50%, score 3 = 51–75%, score 4 = 76–100%) of the tumor area. Tissues from testis served as positive control. For negative controls, testis tissue and glioblastoma sections were stained only with the secondary antibody (Supplementary Fig. 1a) [22].

#### Survivin staining

Immunostaining for survivin was performed on 4-µm sections of formalin-fixed paraffin-embedded tissue using the Leica Bond-Max automated immunostaining system (Leica Biosystems AB, Kista, Sweden). For antigen retrieval, samples were incubated for 20 min at 100 °C with Bond Epitope Retrieval Solution 1 (Leica Biosystems). Slides were stained for 30 min at room temperature with the survivin polyclonal antibody RB-9245 (Thermo Scientific), diluted 1:200. The percentage of positive cells was evaluated using a semiquantitative score: 1 + < 10%, 2 + = 10–20%, 3 + = 20–50% and 4 + > 50% of the tumor area (Supplementary Fig. 1b).

#### Whole-blood assay (WBA) and IFN-γ ELISA

Whole blood was obtained at the time of diagnosis from patients with gliomas (i.e., prior to surgery, radiation and chemotherapy), diluted 1:1.5 with RPMI 1640 l-glutamine (2 mM) with antibiotics (penicillin, 100 IU/mL, and streptomycin, 100 µg/mL) (Life Technologies, Carlsbad, USA) and incubated (i) without cytokines (RPMI only), (ii) with a IL-7 (10 ng/ml)/IL-2 (500 IU/ml) cytokine cocktail or (iii) with a IL-2 (1000 IU/ml)/IL-15 (10 ng/ml)/IL-21 (10 ng/ml) cytokine cocktail (Prospec, Ness Ziona, Israel). The diluted blood was co-incubated in pre-coated plates with a panel of different TAA and viral antigens (Supplementary Table 1) for 7 days at 37 °C and 5% CO_2_ as described previously [23, 24]. To gauge the basal IFN-γ production to NY-ESO-1, survivin as well as commonly recognized target antigens, blood was incubated with medium (negative control) and non-tumor-related antigens, i.e., the Epstein–Barr virus nuclear antigen 3 (EBNA-3). Positive controls were phytohemagglutinin (PHA, Sigma-Aldrich), OKT3 (anti-CD3 monoAb, Biolegend, CA, USA, 30 ng/ml) and SEA + SEB (staphylococcal enterotoxin A and B). IFN-γ production was then quantified in the cell culture supernatant by ELISA (Mabtech, Stockholm, Sweden).

#### FASCIA CD3^+^/CD4^+^/CD8^+^ proliferation

On day 7, cell culture supernatants were harvested, and blood of the duplicate wells was collected and washed with phosphate-buffered saline (PBS). Cells were then stained with the flow-cytometric assay of specific cell-mediated immune response in activated whole blood (FASCIA) test [25] with a cocktail of monoclonal surface antibodies anti-CD3 FITC, anti-CD4 APC and anti-CD8 PerCP. After incubation of 15 min at +4 °C, red blood cells were lysed using a Pharm lysing buffer (BD Biosciences, CA, USA) for 10 min, and cells were then incubated for 5 min at room temperature. The immune cells were then resuspended in PBS and analyzed using a FACSCalibur flow cytometer (BD Biosciences, CA, USA) and FlowJo analytical software (Treestar, OR, USA). The proliferation ratio was calculated with resting and activated cells (blasts) according to their size and granularity: PR = blast/(resting cells + blast). The stimulation percentage (SP%) was defined as the negative (medium) and positive (PHA) control proliferation ratio: SP*%* = (PR_Ag_ − PR_medium_)/(PR_PHA_ − PR_medium_) × 100 as described in detail previously [25].

#### Qualitative NY-ESO-1 and survivin IgG ELISA

Plasma was obtained from patients with glioblastoma at the time of diagnosis, prior to surgery (and to radiation/chemotherapy), and stored at −20 °C. Plasma levels of antibodies against NY-ESO-1 or survivin were determined using an indirect IgG ELISA. Ninety-six-well plates (NUNC, Roskilde, Denmark) were coated with human IgG as a reference standard (ranging between 1875 and 15 ng/ml for NY-ESO-1 assay and between 500 and 3,9 ng/ml for the survivin assay, Sigma-Aldrich, St. Louis, MI, USA) in a seven-point serial dilution (1:2 ratio); other wells contained the respective protein antigens (NY-ESO-1 and survivin, MyBioSource, San Diego, USA). The plate was incubated for 1 h at 37 °C, washed three times with washing buffer (PBS 0.05% Tween 20, Sigma-Aldrich, St. Louis, MI, USA) and then blocked with PBS 2% BSA 0.05% Tween 20 buffer, followed by incubation at room temperature for 1 h and five subsequent washing steps. Diluted patient samples were then incubated in duplicates for 2 h at room temperature (RT) and washed five times afterward. The plate was incubated with a secondary antihuman IgG, mAb (ALP conjugated, 1:1000 dilution, Mabtech, Stockholm, Sweden) for 1 h at RT and washed five times. Para-nitrophenyl phosphate (pNPP, Thermo Fisher Scientific, MA, USA) was added to the plate for 45 min at RT in the dark, and the reaction was stopped by adding 1 N NaOH. The optical density was measured at 405 nm and the anti-specific reactivity expressed as IgG equivalents.

### PBMCs isolation and culture

PBMCs (peripheral blood mononuclear cells) were isolated from heparinized blood over a Ficoll–Hypaque gradient as described previously [26] prior to surgery, radiation and chemotherapy and preserved at −190 °C using 90% fetal bovine serum (FBS, Life Technologies, Carlsbad, USA) and 10% DMSO. For T-cell expansion, PBMCs were cultured freshly after separation in CellGro (serum-free medium CellGenix, Freiburg, Germany) with 5% pooled human AB serum (Innovative Research, Michigan, USA), supplemented with recombinant IL-2 (1000 IU/ml)/IL-15 (10 ng/ml)/IL-21 (10 ng/ml) (Prospec, Ness Ziona, Israel) in six-well plates (BD Falcon) at 2 million cells/mL for 7 days with the respective antigens (NY-ESO-1 peptide mix or the survivin peptide mix; Peptides&Elephants, Potsdam, Germany; the peptide concentration was 1 µg/ml for each peptide). At day 7, cells were restimulated with irradiated autologous PBMCs at ratio 1:10, loaded with NY-ESO-1 or survivin peptides mix (at 1 µg/peptide/ml) in the presence of cytokines as listed above. At day 10, OKT3 (anti-CD3 monoAb, Biolegend, CA, USA) was added at 30 ng/mL to the cell culture in order to amplify the specific immune response. Cells were harvested at day 18 and analyzed. If necessary, the culture medium was changed by replacing half of the culture volume. The targets were: NY-ESO-1 (SWISS PROT P78358) represents a peptide mix of 42 × 15mer peptides with an 11aa overlap which covers the entire length of the NY-ESO-1 sequence (180 aa); the survivin (SWISS PROT O15392) peptide mix represents 33 × 15mer peptides with an 11aa overlap covering the entire length of the survivin sequence (142 aa). Expanded T-cells were tested after harvest at day 18. After a single wash in PBS, 1 million cells were stained with the following Ab cocktail: anti-CD3 Percp (BD Biosciences, CA, USA), anti-CD4 Krome Orange (Beckman Coulter, CA, USA), anti-CD8a APC-Cy7 (BD Biosciences, CA, USA), anti-CD8β FITC (Beckman Coulter, CA, USA), anti-CD45RA ECD (Beckman Coulter, CA, USA) and anti-CCR7 Brilliant Violet 421 (Biolegend, CA, USA).

### Immunophenotype, T-cell activation panel and tetramer-guided T-cell analysis

One million T-cells were stained with the following Ab cocktail: anti-CD3 Brilliant Violet 570 (Biolegend, CA, USA), anti-CD4 Brilliant Violet 510 (Biolegend, CA, USA), anti-CD8a APC-Cy7 (BD Biosciences, CA, USA), anti-4-1BB FITC (eBioscience, CA, USA), anti-CD127 APC-AF700 (Beckman Coulter, CA, USA), anti-CD45RA ECD (Beckman Coulter, CA, USA), anti-CCR7 Brilliant Violet 421 (Biolegend, CA, USA), anti-LAG-3 APC (R&D Systems, Minneapolis, MN), anti-CD25 PE-Cy7 (BD Biosciences, CA, USA), anti-CTLA-4 PE-Cy5 (BD Biosciences, CA, USA), anti-TIM3 Percp-eFluor710 (eBioscience, CA, USA) and anti-PD-1 PE (BD Biosciences, CA, USA). After 15 min, T-cells were washed with PBS–0.1% FBS and analyzed using a FACSAria flow cytometer (BD Biosciences, Stockholm, Sweden); data analysis was performed using FlowJo software.

### NY-ESO-1 tetramer

PBMCs from HLA-A*0201-positive patients with GBM were stained with the HLA-A*02:01-restricted NY-ESO-1 antigen SLLMWITQV-PE (10 μL, Immudex, Copenhagen, Denmark) for 30 min at 37 °C prior to washing with PBS–0.1% FBS, followed by staining with the cell surface antibody cocktail described above.

### Tumor cell line culture and decitabine treatment

Two glioma cell lines (DBTRG05-MG and SNB19) and the melanoma M624 tumor cell line were maintained in RPMI 1640 l-glutamine (2 mM) with antibiotics (penicillin, 100 IU/mL, and streptomycin, 100 µg/mL) and 10% FBS (Life Technologies, Carlsbad, USA). The HLA-A2^+^ glioma tumor and melanoma cell lines were treated with decitabine (Selleckchem, Houston, USA) at 1 µM as described previously [27].

### Real-time PCR analysis of NY-ESO-1 expression

Total RNA was extracted from DBTRG05-MG and SNB19 and M624 using the AllPrep DNA/RNA Mini Kit (Qiagen Inc., Hilden, Germany) according to the supplier’s instructions. cDNA synthesis and real-time PCR analysis of NY-ESO-1 expression in tumor cell lines were performed as described previously by Odunsi K et al. [28].

### NY-ESO-1-specific T-cell isolation and T-cell tumor co-culture

Two different methods were used in order to isolate NY-ESO-1-specific T-cells from PBMCs of two HLA-A2+ patients with glioma after expansion as described earlier. (i) After expansion, cells were stained with the NY-ESO-1 HLA-A2 dextramer reagent (Immudex, Denmark) for 30 min at RT and then stained with cell surface markers: ECD anti-CD3 Ab (Beckman Coulter, CA, USA), V450 anti-CD4 Ab (BD Biosciences, CA, USA) and the Percp anti-CD8 Ab (BD Biosciences, CA, USA) for 15 min at 4 °C. (ii) NY-ESO-1 HLA-A2 dextramer-specific cells were sorted using an Aria flow cytometer (BD Biosciences, Stockholm, Sweden). After NY-ESO-1 peptide-driven expansion, T-cells were stimulated with the NY-ESO-1 peptide mix (Peptides&Elephants, Custom Product) for 6 h at 37 °C, 5% CO_2_, and NY-ESO-1-specific T-cells were purified with the IFN-γ Production Assay Cell Enrichment and Detection Kit (PE) (Miltenyi Biotec, CA, USA) according to the supplier’s instructions. After separation, NY-ESO-1-specific T-cells were exposed to tumor cells (± decitabine pretreatment) in duplicates in 96-well plates and T-cell responses were blocked with W6/32 (anti-HLA-A, B, C; Sigma-Aldrich, St. Louis, USA) or L243 (anti-HLA-DR; Biolegend, San Diego, CA, USA) at 1 μg/well. T-cells were cultured at a E/T ratio of 4:1 for T-cells from dextramer-sorted T-cells and at 5:1 for T-cells isolated by IFN-γ capture. Supernatants were harvested at day 3 and tested for IFN-γ by ELISA (Mabtech, Stockholm, Sweden).

### Statistical analysis

Differences between patient groups (i.e., G versus A versus OA/O or 4 versus 3 versus 2) or within each group (i.e., comparing medium culture versus cultures with antigens) were analyzed using the Mann–Whitney *U* test or the Wilcoxon test using GraphPad Prism 6 software. The Spearman test and linear regression analysis with the GraphPad software were used to test the correlations.

## Results

### Survivin and NY-ESO-1 protein expression in gliomas

**Fig. 1:**
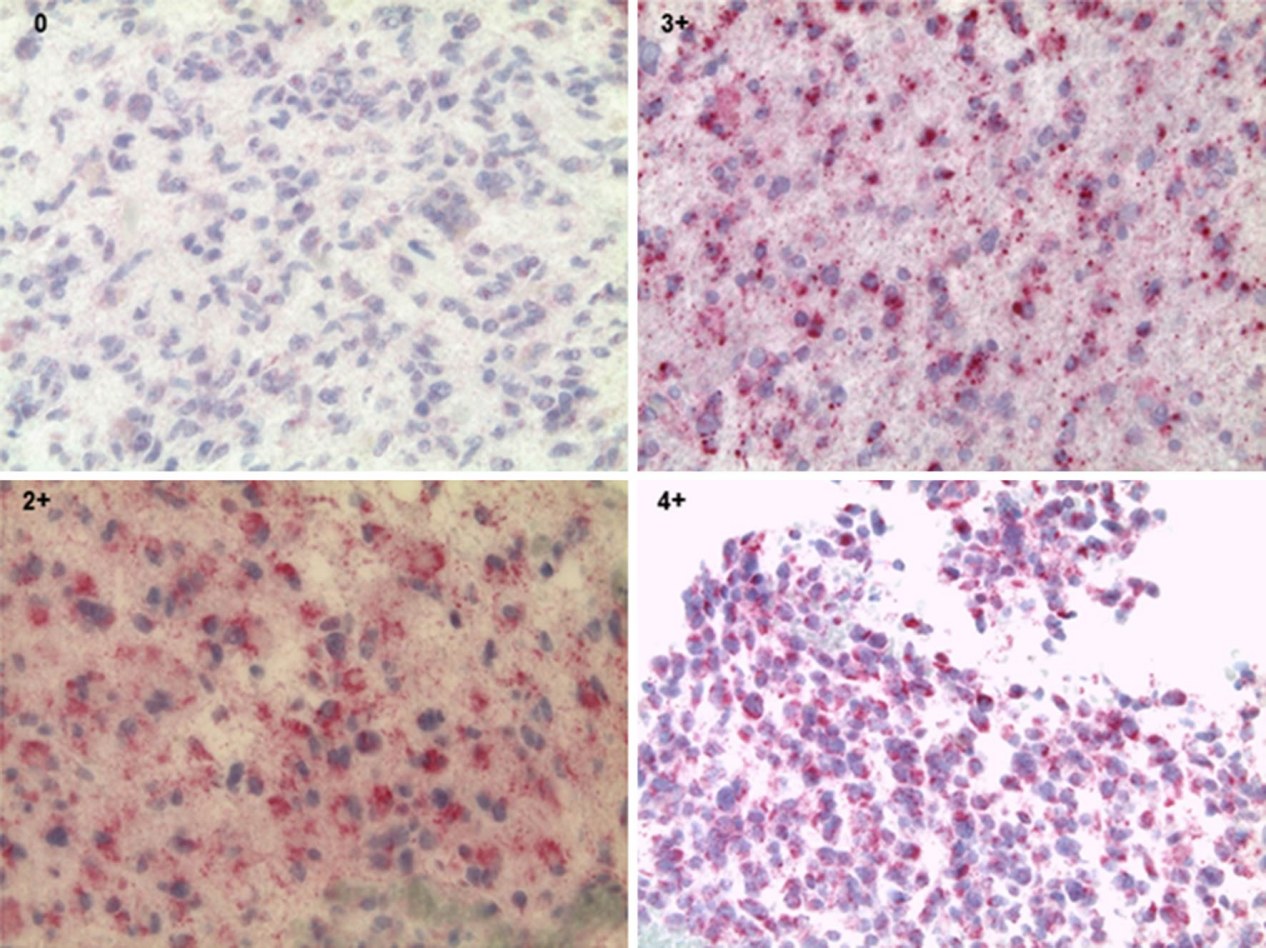
Immunohistology of NY-ESO-1, magnification ×40. Each tissue section was semiquantitatively scored based on the intensity of immunostaining: 0 = tumor cells stain negative. Positive: score 2 = 26–50%, score 3 = 51–75%, score 4 = 76–100% of the tumor area

Tumor sections were tested for NY-ESO-1 and survivin protein expression (Fig. 1 and Supplementary Fig. 1a, b). In 40 sample sections, analyzed for survivin, 22.5% (9/40) scored 4+, 27.5% (11/40) scored 3+, 25% (10/40) scored 2+ and 25% (10/40) scored 1+ (Supplementary Table 2). Most of the ‘high score’ sections testing positive for survivin represent grade IV glioma (18/20). Twenty-eight out of 38 GBM specimens tested positive for NY-ESO-1: 5.26% (2/38) scored as 4+, 2.63% (1/38) exhibited a score of 3+, 7.89% (3/38) tested for 2+, 23.68% (9/38) of samples tested 1+, 34.21% (13/38) of samples exhibited focal staining and 26.31% (10/38) of samples tested negative (Table 2 and Supplementary Table 2).

**Table 2:** NY-ESO-1 and survivin protein expression

Diagnosis	Grade	Survivin score (*n* = 40)				NY-ESO-1 score (*n* = 38)					
		1+	2+	3+	4+	Neg	Focal	1+	2+	3+	4+
Astro/GBM	2	4	2			1	2	2		1	
	3	2							1		
	4	3	5	9	9	7	10	5	2		1
Oligo/mixed	2	1	1					2			
	3		2	2		2	1				1
Total	10	10	11	9	10	13	9	3	1	2	

### IFN-γ production and cellular proliferation in response to TAAs

**Fig. 2:**
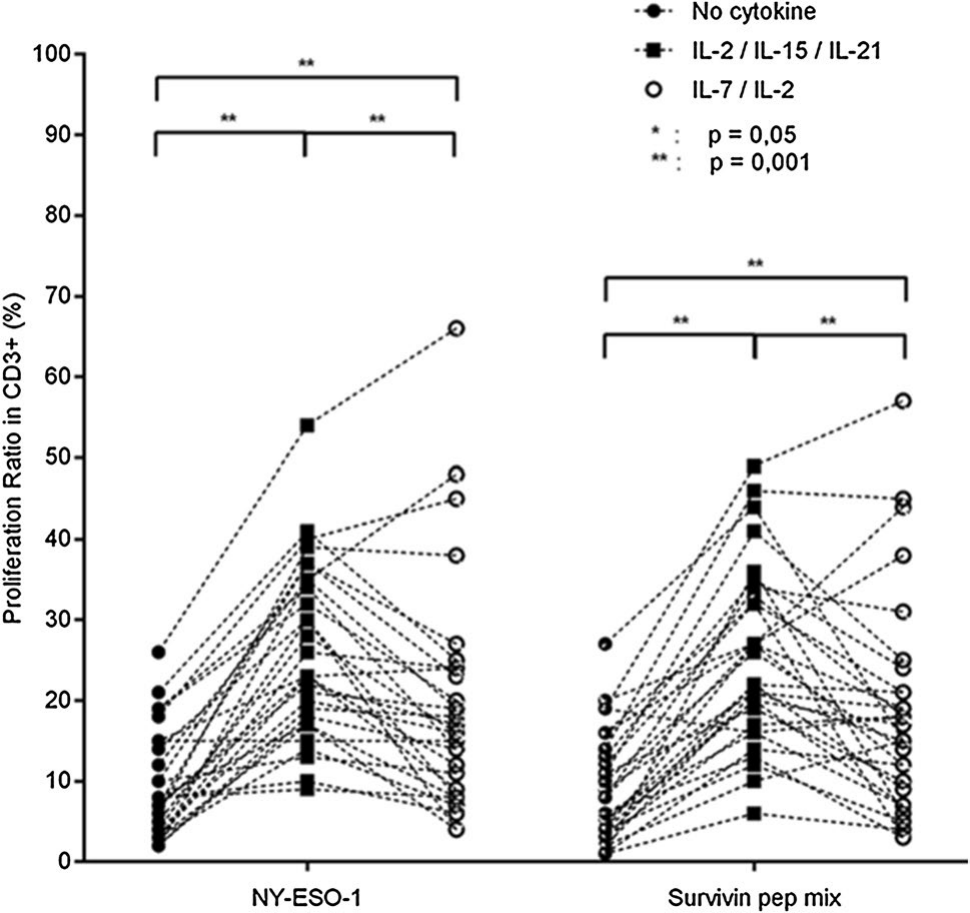
T-cell proliferation ratio after a 7-day expansion of peripheral blood with NY-ESO-1 or the survivin peptide mix. Three different conditions: (i) without cytokines (RPMI only), (ii) with a IL-7/IL-2 cytokine cocktail or (iii) with a IL-2/IL-15/IL-21 cytokine cocktail (*p ≤ 0.05, **p ≤ 0.001)

NY-ESO-1 and survivin were tested to drive cellular proliferation and IFN-γ production in blood from patients with GBM, as well as to control antigens, including frequently recognized Flu targets (H1N1, H1N5), as well as to negative controls, e.g., *Mycobacterium tuberculosis* targets (Supplementary Figs. 2a, b and 3). We identified an association of TAA-reactive T-cells (defined by IFN-γ production) in correlation with the histopathological grading of the tumor and T-cells cultured with IL-2/IL-15 and IL-21. Stronger IFN-γ production was identified in PBMCs from patients with histopathological grade III tumors as compared to patients with a grade IV tumor in response to NY-ESO-1 (*p* = 0.0135); this observation was also found to be true for IFN-γ production to the survivin peptide mix (*p* = 0.0062, Supplementary Fig. 4). The cellular proliferation ratio was increased using IL-2/IL-15/IL-21 as compared to IL-2/IL-7-driven T-cell expansion for the antigen NY-ESO-1 (*p* = 0.0014) (Fig. 2). We did not observe differences concerning the proliferative index between the IL-2/IL-7 and IL-2/IL-15/IL-21 cytokine cocktails for survivin-driven T-cell expansion. Of note, the IL-2/IL-15/IL-21 cytokine combination particularly increased the CD8^+^ T-cell population as compared to other culture conditions (IL-2/IL-7 or medium without cytokines) in response to the survivin peptide mix (*p* = 0.0013).

### IFN-γ production in response to individual TAA peptides

**Fig. 3:**
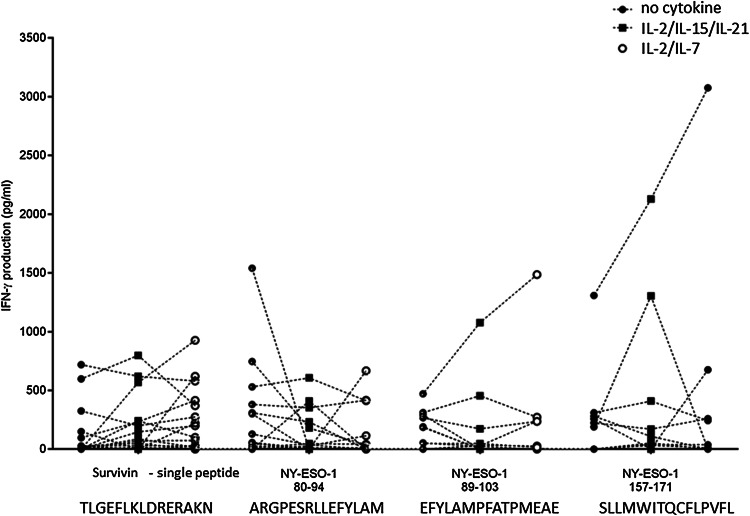
IFN-γ production after a 7-day expansion of peripheral blood with single TAA peptide antigens that have been shown to be immunodominant (survivin 97–111, the peptides NY-ESO-1 80–94, 89–103 and 157–171); three different conditions: (i) without cytokine (RPMI only), (ii) with a IL-7/IL-2 cytokine cocktail or (iii) with a IL-2/IL-15/IL-21 cytokine cocktail. Data shown after subtraction of the constitutive IFN-γ production

TAA-driven T-cell expansion was tested with peptides covering the entire NY-ESO-1 or the survivin protein. We tested additionally single peptides from survivin and from NY-ESO-1 (see “Materials and methods” section) that have previously been reported as ‘hot spots’ for immunodominant T-cell recognition. The subsequent T-cell response was measured by IFN-γ production, and cellular proliferation was evaluated after a 7-day incubation. We did not identify significant differences among the three different culture conditions (no cytokines, IL-2/IL-7 or IL-2/IL-15/IL-21) concerning TAA-driven expansion of lymphocytes using a single survivin peptide, or individual peptides derived from NY-ESO-1, i.e., peptides NY-ESO-1 80–94 or 89–103 or 157–171 (Fig. 3). We observed stronger T-cell reactivity, using IL-2/IL-15/IL-21, defined by IFN-γ production in blood from patients with grade III glioma as compared to blood from patients with grade II glioma (*p* = 0.045) using a single peptide epitope from survivin that has previously been reported to be immunodominant and to be presented by a broad range of MHC alleles [29] (Supplementary Fig. 4).

### Humoral immune responses against TAAs

Specific IgG against TAAs from patients with glioma was compared with IgG obtained from healthy donors (matched for age and gender). The humoral response against NY-ESO-1 was found to be significantly higher among patients with glioma as compared to anti-NY-ESO-1 IgG responses found in the age- and sex-matched (healthy) donors. The specific anti-survivin IgG among patients with glioma exhibited a stronger response as compared to the humoral response from healthy donors against survivin. A correlation analysis of immunohistology with humoral response is reported in detail in Supplementary Fig. 5.

### Expansion of antigen-specific T-cells from PBMCs

In order to evaluate the cytokine production at a single-cell level, we expanded PBMCs (after Ficoll separation) from five patients with the NY-ESO-1 or the survivin peptide mix in the presence of IL-2/IL-15/IL-21 and tested T-cell maturation (based on CD45RA/CCR7 marker expression, Supplementary Fig. 6) and T-cell activation, including 4-1BB. NY-ESO-1- or survivin-driven T-cell expansion resulted in different frequencies of antigen-specific CD4^+^ and CD8^+^ T-cells (defined by 4-1BB reactivity) ranging from 2.78 to 26.5% CD4^+^ T-cells (Supplementary Table 3 and Supplementary Fig. 7). NY-ESO-1-specific T-cell responses, i.e., cytokine production defined by ICS, were detectable in T-cells from patients whose tumor lesions stained positive for NY-ESO-1 protein expression (Supplementary Table 4). Anti-NY-ESO-1 reactivity was confirmed by MHC class I (HLA-A2^+^)-peptide-tetramer-guided staining showing up to 9.25% HLA-A2 + (NY-ESO-1)-reactive T-cells (Supplementary Fig. 8). PBMCs (50 × 10e6 cells as the starting cell numbers) could consistently be expanded (PBMCs from *n* = 5 individual patients with GBM) to more than 2 × 10e8 T-cells (data not shown).

### NY-ESO-1 GBM tumor cells are recognized by sorted NY-ESO-1-specific T-cells

In order to show that NY-ESO-1 (peptide)-expanded T-cells recognize naturally and presented tumor-associated peptides, we used decitabine that induces NY-ESO-1 expression in cell lines. GBM cell lines (± decitabine), or a NY-ESO-1 + HLA-A2^+^ melanoma cell, were used as target cells and co-cultured with NY-ESO-1-specific T-cells isolated from two HLA-A2^+^ glioma patients (either by tetramer-guided sorting, or by a cytokine capture assay, Supplementary Fig. 9). IFN-γ production in NY-ESO-1+-sorted T-cells showed that NY-ESO-1-peptide-expanded T-cells were able to react against naturally processed and presented peptides on HLA-A2^+^ tumor cell lines.

## Discussion

We tested NY-ESO-1 and survivin expression in gliomas and analyzed the corresponding patient’s PBMCs for anti-TAA reactivity. NY-ESO-1 expression in glioma was found to be low in a single study [30] as compared to the current report. A possible explanation for this difference may be the selection of patients. The patients in our cohort presented with a primary tumor prior to radiation or any chemotherapy [31]. NY-ESO-1 expression was also found to be patchy (this report); limited access to tumor material may therefore result in false negative results concerning protein expression. NY-ESO-1 and survivin expression was further consolidated by the presence of humoral anti-NY-ESO-1- and survivin-directed IgG responses [32, 33] in the current patient cohort (see Supplementary Fig. 5). NY-ESO-1 protein expression in glioma may therefore open new therapeutic options, given the recent success of anti-NY-ESO-1-directed transgenic TCRs (for HLA-A2^+^) individuals [34, 35], the use of anti-NY-ESO-1-directed antibody therapies [36] or the use of anti-NY-ESO-1-directed vaccination strategies [37].

The proliferative capacity of PBMCs in response to TAAs suggests that NY-ESO-1- or survivin-directed T-cells can be expanded and used for the cellular therapy of patients with glioma: tetramer-guided or IFN-γ-captured NY-ESO-1-directed T-cells were shown to recognize naturally processed and presented epitopes, suggesting that peptide-driven expansion of T-cells leads to biologically and clinically relevant T-cell populations directed against tumor cells (see Supplementary Fig. 8).

The number of cellular therapies directed against tumor-associated antigens for patients with gliomas is limited up to now; a review of cell-based therapies suggests that infusion of immune cells may lead to improve survival along with limited therapy-associated toxicity. For instance, PBMCs were harvested for cellular therapy and CTL were generated directed against autologous (glioma) tumor cells (using a mix of PBMCs, autologous tumor cells and recombinant IL-2), followed by in situ administration (10^8^ up to 10^9^ T-cells i.t.). Three out of five patients did not exhibit any benefit; 1/5 patient showed a transient regression; and 1/5 patient showed a complete regression that lasted 104 weeks [38]. TAAs could provide an ‘off-the-shelf solution’ to drive anticancer-directed T-cells. Alternate methods have been reported, i.e., (i) OKT3 stimulation of peripheral T-cells along with IL-2 was used to treat nine patients with glioma. Two out of nine patients (grade III glioma) experienced partial regression that lasted five years [39]. (ii) A different approach was the use of T-cells present in regional lymph nodes for adoptive therapy [40]. Two out of nine patients showed tumor regression, one of them a durable response that lasted 17 months. (iii) Treatment with LAK cells (lymphokine-activated killer cells) [41]: A single study was performed in patients with glioma after surgical resection, followed by intralesional application of LAK cells with a median survival of 53 weeks as compared to 25.5 weeks of the control group [42]. Dillmann and coworkers showed in phase II study a 25-month median survival and a 75% 1-year survival in 40 patients with intralesional LAK cells; this study was performed in patients with newly diagnosed glioma [43]. Infusion of TIL (*n* = 6) obtained from patients with glioma suggested a clinically relevant benefit for individual patients with a complete response [44]. The data in our report show that NY-ESO-1 and survivin can now be added as a tumor-associated target for the biological treatment of patients with glioma, particularly since data from several NY-ESO-1-directed [45] or anti-survivin-directed trials did not report [46] major toxicity.

The presence of not only anti-tumor-directed T-cells, but also their phenotypic profile and homing pattern appears to be responsible for clinical efficacy. Clinical experience from patients with melanoma showed that (tumor) antigen-specific T-cells persist, acquire a central memory phenotype and are able to mediate long-term (up to 3 years) remissions in some patients [47]. The CD45RA-CCR7^+^ central memory T-cell phenotype appears to be a key for long-lasting anti-tumor-directed cellular immune responses [48]. This T-cell phenotype may also be achieved expanding anti-NY-ESO-1- or survivin-directed T-cells, followed by infusion into patients with NY-ESO-1^+^ or survivin + gliomas. Of note, the CD45RA-CCR7^+^ central memory T-cell subset population, associated with increased responsiveness to therapy, was not depleted upon IL-2/IL-15/IL-21-driven expansion (see Supplementary Fig. 6).

A matter of discussion may be the absolute number of antigen-specific T-cells required for passive transfer of antigen-specific T-cells. Even low numbers of anti-TAA-directed T-cells may be able to mediate clinically relevant effects, since (i) conditioning of the patients, prior to T-cell infusion (using fludarabine and cyclophosphamide), will provide ‘space’ for T-cell expansion after adoptive transfer and upon encounter with the nominal target antigen expressed by the tumor, (ii) IL-21 in concert with IL-15 (cytokines used in the current report) prevents T-cell apoptosis of immune cells responding to antigenic stimulation [49] and (iii) data from experimental models suggest that low numbers of precursor T-cells are able to expand into effector T-cell populations [50]. We could also show that T-cells directed against NY-ESO-1 can be expanded from PBMCs obtained from healthy individuals (see Supplementary Table 5). The fact that low (absolute) numbers of TAA-reactive immune cells are able to mediate biologically relevant effects is supported by data after allogeneic stem cell transplantation, i.e., even low numbers of (tetramer-sorted) antigen (CMV)-specific T-cells, as low as 10.000 cells/kg patient, are able to confer protective, clinically relevant target-specific cellular immune responses [51]. The fact that antigen-specific IFN-γ responses could be detected in 25% blood samples for NY-ESO-1 and in 30% for survivin suggests that there is a viable TCR repertoire present capable of reacting to these nominal TAAs. Further studies are needed to examine whether (i) the extent of NY-ESO-1 or survivin protein expression is associated with increased numbers of antigen-specific T-cells and (ii) the TCR repertoire in ‘non-responding’ patients has been depleted due to prolonged antigen exposure.

### Electronic supplementary material

Below is the link to the electronic supplementary material.

Supplementary material 1 (PDF 3068 kb)

## Abbreviations

A: Astrocytoma
EBNA-3a: Epstein–Barr virus nuclear antigen 3
FASCIA: Flow cytometric assay of specific cell-mediated immune response in activated whole blood
GBM: Glioblastoma multiforme
ICS: Intracellular cytokine staining
IFN-γ: Interferon gamma
IL-2: Interleukin-2
OA/O: Oligoastrocytoma/oligodendroglioma
PBMCs: Peripheral blood mononuclear cells
PHA: Phytohemagglutinin
PR: Proliferation ratio
TAA: Tumor-associated antigen
TCR: T-cell receptor
TNF-α: Tumor necrosis factor alpha
RT: Room temperature
