# Supplementary material for: NY-ESO-1- and survivin-specific T-cell responses in the peripheral blood from patients with glioma
Source: Cancer Immunol Immunother. 2017 Oct 20;67(2):237–46. doi: 10.1007/s00262-017-2066-z (PMC5799356; doi:10.1007/s00262-017-2066-z)
Supplement: Supplementary file 1 — Supplementary material 1 (PDF 3068 kb) [file 262_2017_2066_MOESM1_ESM.pdf]

SUPPLEMENTARY TABLES 1-5

Supplementary Table 1: List of antigens used for the Whole Blood Assay (WBA)

Antigen	Type	Final concentration	Access NO.
PHA	protein	5µg/ml	L901710MG
CD3 Ab	protein	30ng/ml	Clone:OKT3
SEA+SEB	protein	10ng/ml	SEA:Sigma#9399 SEB:S-4481
EBNA-1	protein	1µg/ml	P03211 (swiss prot)
EBNA-3a	protein	1µg/ml	P12977 (swiss prot)
A-VIET(H5N1)	protein	2.5µg/ml	Baxter (HA protein)
H1N1 California	protein	2.5µg/ml	GSK split
ESAT-6	protein	1µg/ml	AEP68523
CMV-pp65	protein	1µg/ml	P06725 (swiss prot)
EGFRvIII	peptide	1µg/ml	LEEKKGNYVVTDH
NY-ESO-1	peptide mix	1/peptide/ml	P78358 (swiss prot)
NY-ESO-1 80-94	peptide	1µg/ml	ARGPESRLLEFYLAM-NH2
NY-ESO-1 89-103	peptide	1µg/ml	H- EFYLAMPFATPMEAE- NH2
NY-ESO-1 157-171	peptide	1µg/ml	SLLMWITQCFLPVFL-NH2
survivin single peptide	peptide	1µg/ml	TLGEFLKLDRERAKN
survivin peptide mix	peptide mix	1/peptide/ml	Q5RAH9 (swiss prot)

Supplementary Table 2: Details of the immunohistology staining results for NY-ESO-1 and survivin (n.d. = not detetermined).

Study ID	Diagnosis / Grade	Survivin-score	Immunohistology Staining NY-ESO-1
**GBM 1**	GBM / IV	2+	Focal
**GBM 2**	GBM / IV	3+	2+
**GBM 4**	GBM / IV	3+	Focal
**GBM 5**	GBM / IV	2+	Neg
**GBM 6**	GBM / IV	4+	Focal
**GBM 7**	Astro / II	2+	Focal
**GBM 8**	Astro / III	1+	2+
**GBM 9**	Oligo / II	1+	1+
**GBM 10**	GBM / IV	2+	1+
**GBM 12**	GBM / IV	3+	1+
**GBM 13**	GBM / IV	1+	n.d.
**GBM 14**	Astro / III	1+	n.d.
**GBM 15**	Astro / II	1+	3+
**GBM 16**	Astro / II	1+	1+
**GBM 17**	Astro / II	2+	Neg
**GBM 18**	GBM / IV	4+	1+
**GBM 19**	GBM / IV	3+	Focal
**GBM 20**	GBM / IV	4+	Neg
**GBM 21**	OA / II	2+	1+
**GBM 22**	Oligo / III	2+	Neg
**GBM 23**	GBM / IV	4+	1+
**GBM 24**	GBM / IV	1+	1+
**GBM 25**	GBM / IV	2+	Focal
**GBM 26**	GBM / IV	2+	Focal
**GBM 27**	OA / III	3+	Focal
**GBM 28**	OA / III	2+	4+
**GBM 29**	GBM / IV	3+	Focal
**GBM 30**	GBM / IV	4+	Focal
**GBM 31**	GBM / IV	3+	4+
**GBM 32**	GBM / IV	4+	Focal
**GBM 33**	GBM / IV	3+	Neg
**GBM 35**	Astro / II	1+	Focal
**GBM 36**	GBM / IV	4+	Neg
**GBM 37**	GBM / IV	4+	Neg
**GBM 38**	Oligo / III	3+	Neg
**GBM 39**	GBM / IV	4+	Neg
**GBM 40**	GBM / IV	3+	Focal
**GBM 41**	Astro / II	1+	1+
**GBM 42**	GBM / IV	1+	2+
**GBM 43**	GBM / IV	3+	Neg

Supplementary Table 3: Antigen-driven expression of activation markers in PBMCs from patients with GBM

Activation marker		Ag stimulation	GBM 30 *T0*	GBM 30 Ag	GBM 31 *T0*	GBM 31 Ag	GBM 33 *T0*	GBM 33 Ag	GBM 34 *T0*	GBM 34 Ag	GBM 35 *T0*	GBM 35 Ag
CD4+	**CD4+**	**EGFRVIII**		12,2		39		10,9		44,3		74,4
		**NY-ESO-1**	*40,4*	30,5	*70*	62,5	*36,2*	30,6	*48,8*	63,1	*65,6*	74,8
		**Survivin**		24,8		70,5		33,5		63,7		72,2
	**4-1BB+**	**EGFRVIII**		1,39		1,85		0,39		0,91		1,47
		**NY-ESO-1**	*0,13*	26,5	*0,16*	50,2	*0,032*	16,9	*0,28*	3,47	*0,083*	2,78
		**Survivin**		34,2		43,4		14,6		11,9		4,02
	**CD25+**	**EGFRVIII**		31,8		50,6		12		84,3		86,9
		**NY-ESO-1**	*2,76*	66	*6,43*	85,7	*4,01*	29,6	*10,1*	60,5	*4,73*	97,5
		**Survivin**		56,4		88,2		30,6		76		94,2
	**CD127+**	**EGFRVIII**		77,5		34,7		84,9		16,4		8,08
		**NY-ESO-1**	*77,6*	60,4	*69,5*	49	*88,5*	64,3	*35,7*	34,3	*42*	2,08
		**Survivin**		55,8		54		71,5		35,8		6,77
	**CTLA-4+**	**EGFRVIII**		0,88		1,79		0,44		1,19		3,82
		**NY-ESO-1**	*0,27*	6,87	*1,04*	7,59	*0,19*	0,93	*1,64*	3,03	*1,28*	3,66
		**Survivin**		4,91		7,43		1,46		7,28		7,04
	**LAG3+**	**EGFRVIII**		7,71		10,1		4,68		2,73		3,06
		**NY-ESO-1**	*10,7*	20,8	*12,5*	26,3	*4,05*	10,8	*3,66*	4,65	*2,21*	2,58
		**Survivin**		20,5		17		13,9		5,98		3,79
	**PD-1**	**EGFRVIII**		21,9		34,2		9,13		29,3		84,5
		**NY-ESO-1**	*7,1*	55,8	*37,8*	50,2	*14,8*	15,1	*3,99*	39,4	*4,64*	87,2
		**Survivin**		47,1		55,4		17,2		55,8		90,5
	**TIM3+**	**EGFRVIII**		0,066		0,056		0,016		0,35		0,75
		**NY-ESO-1**	*0*	0,34	*0*	0,36	*0*	0,063	*0,13*	0,52	*0,044*	0,066
		**Survivin**		0,28		0,27		0,5		1,71		0,21

Activation marker		Ag stimulation	GBM 30 *T0*	GBM 30 Ag	GBM 31 *T0*	GBM 31 Ag	GBM 33 *T0*	GBM 33 Ag	GBM 34 *T0*	GBM 34 Ag	GBM 35 *T0*	GBM 35 Ag
**CD8+**	**CD8+**	**EGFRVIII**		34,7		30,8		25,3		34,9		16
		**NY-ESO-1**	*18,2*	33,9	*19,6*	9,63	*39,6*	33,2	*20,1*	25,5	*9,55*	13,2
		**Survivin**		36,2		11,1		45		22,2		15,1
	**4-1BB+**	**EGFRVIII**		0,78		0,49		0,47		0,96		1,93
		**NY-ESO-1**	*1,64*	13,3	*1,16*	24,8	*0,66*	12,5	*0,067*	7,57	*0,17*	10,6
		**Survivin**		12,4		25,2		11		16,2		11,6
	**CD25+**	**EGFRVIII**		59,3		44,1		25,8		90,1		86,9
		**NY-ESO-1**	*5,58*	43	*5,98*	97,6	*1,59*	65,2	*3,47*	50,9	*1,4*	96
		**Survivin**		45,4		94,6		44,4		65		94,4
	**CD127+**	**EGFRVIII**		32,6		30		43,6		15,6		21,6
		**NY-ESO-1**	*68,8*	47,6	*62,7*	57,1	*67,4*	49,4	*42,5*	37	*52,6*	12,1
		**Survivin**		42,9		53,1		57,1		37,1		19,6
	**CTLA-4+**	**EGFRVIII**		1,17		1,48		1,62		8,46		24,2
		**NY-ESO-1**	*1,32*	3,58	*2,16*	8,89	*1,37*	2,08	*12*	19,4	*14,3*	15,6
		**Survivin**		2,91		7,09		2,91		18,3		28
	**LAG3+**	**EGFRVIII**		92,3		94,5		96,1		12,6		26,2
		**NY-ESO-1**	*79*	94,5	*88,3*	93,1	*91,9*	98,3	*15,9*	25,6	*18,9*	14,3
		**Survivin**		95,5		94,6		98,1		26,5		24,6
	**PD-1**	**EGFRVIII**		18,2		39,5		14,4		21,4		56,5
		**NY-ESO-1**	*7,53*	28,3	*26,3*	44	*4,63*	18,2	*6,04*	26,4	*5,34*	80,4
		**Survivin**		29,4		41,9		12,8		37		82,7
	**TIM3+**	**EGFRVIII**		0,21		0,11		0,15		0,69		2,93
		**NY-ESO-1**	*0*	0,061	*0,016*	0,11	*7E-03*	0,27	*0,12*	1,72	*0,13*	1,2
		**Survivin**		0,19		0,2		1,75		4,13		3,96

Activation marker		Ag stimulation	GBM 30 *T0*	GBM 30 Ag	GBM 31 *T0*	GBM 31 Ag	GBM 33 *T0*	GBM 33 Ag	GBM 34 *T0*	GBM 34 Ag	GBM 35 *T0*	GBM 35 Ag
CD4-CD8-	CD4-CD8-	EGFRVIII		52,5		28,7		63,6		16,6		6,28
		NY-ESO-1	*40,7*	30,1	*9,01*	23	*23,7*	32,6	*28,8*	7,34	*23,8*	9,94
		Survivin		33,4		10,9		17,4		9,33		10,3
	4-1BB+	EGFRVIII		0,83		0,87		0,52		1,17		1,92
		NY-ESO-1	*0,25*	13,5	*0,68*	26,4	*0,099*	15,9	*0,18*	12,6	*0,067*	9,42
		Survivin		17,8		19,8		11,3		17,4		10,1
	CD25+	EGFRVIII		25,4		15,8		14,7		78,4		56,9
		NY-ESO-1	*24,8*	15,8	*7,93*	70,7	*0,41*	35,4	*1,28*	24	*0,27*	71,4
		Survivin		17,1		56,8		18		31,9		64
	CD127+	EGFRVIII		17,7		20,8		13,9		2,31		4,75
		NY-ESO-1	*27,2*	35,5	*41,4*	36,3	*39,9*	25,9	*6,37*	13,8	*13,1*	3,48
		Survivin		35,6		46,3		49,4		14		5,71
	CTLA-4+	EGFRVIII		0,41		1,53		0,75		1,66		13,6
		NY-ESO-1	*0,69*	1,97	*3,43*	4,84	*0,87*	1,3	*2,77*	4,03	*2,9*	12,6
		Survivin		1,92		4,94		1,29		5,16		24,3
	LAG3+	EGFRVIII		10,5		12,7		13,9		2,35		5,63
		NY-ESO-1	*6,05*	21,1	*23,8*	28,9	*25,6*	21,4	*6,1*	7,08	*8,16*	3,07
		Survivin		20,6		28,7		15,4		7,44		6,05
	PD-1	EGFRVIII		18,9		10,9		16,6		8,79		51,1
		NY-ESO-1	*4,14*	17,4	*15,6*	16,8	*6,43*	14,2	*1,29*	10,4	*3,77*	52,7
		Survivin		17,6		22,1		7,32		14,2		52,2
	TIM3+	EGFRVIII		0,12		0,062		0,16		0,66		1,92
		NY-ESO-1	*0*	0,17	*0*	0,28	*0*	0,18	*0,07*	1,39	*0,067*	0,9
		Survivin		0,33		0,1		0,29		2,76		2,58

PBMCs from five patients with glioblastoma were expanded in IL-2, IL-15 and IL-21 as described in the material and method section using i) the cytokine cocktail alone (medium control), ii) the cytokine cocktail plus the respective stimulating antigen (EGFRVIII, NY-ESO-1 or

Survivin). T0= time point zero, start of the T-cell expansion; TH= time of T-cell harvest. The numbers at T0 represent the number of CD4+ within CD3+ T-cells (A), CD8+ (B), or DN (CD4-CD8-) T-cells within CD3+ T-cells (C, numbers are in blue). Different numbers of CD4+, CD8+ and DN (CD4-CD8-) CD3+ T-cells associated with the stimulating antigen. A panel of activation markers was tested and the frequency of activation marker positive T-cells within the respective T-cell population (CD4, CD8 or DN) is reported at time point zero and at the time point of harvest. Numbers represent the frequency of activation marker-positive T-cells in the respective CD4+, CD8+ or DN (CD4-CD8-) CD3+ T-cell population. The values of marker-positive T-cells from the cytokine alone (medium control) have been subtracted. Note the patient-to-patient difference and differences in marker-positive T-cells is associated with the nature of the stimulating antigen, e.g. the difference of 4-1BB positive T-cells in CD4+, CD8+ or DN+ T-cells.

Supplementary Table 4: NY-ESO-1-driven expansion of PBMCs from patients with NY-ESO-1+ glioma

			PMA		NY-ESO-1	
			**Before**	**After**	**Before**	**After**
**GBM 30**	**CD3+/ CD4+**	**IFNγ**	6,29	2,02	0,02	0,00
**GBM 31**		**IFNγ**	16,56	1,71	0,00	0,27
**GBM 35**		**IFNγ**	1,13	2,56	0,00	0,03
**GBM 30**		**IL-2**	43,39	10,44	0,11	0,00
**GBM 31**		**IL-2**	21,19	13,26	0,00	2,11
**GBM 35**		**IL-2**	15,34	58,78	0,01	0,00
**GBM 30**		**TNFα**	37,47	10,99	0,48	0,58
**GBM 31**		**TNFα**	36,06	8,24	1,04	0,67
**GBM 35**		**TNFα**	19,54	53,22	0,00	0,46
**GBM 30**	**CD3+/ CD8+**	**IFNγ**	62,35	49,70	0,29	0,84
**GBM 31**		**IFNγ**	72,56	56,57	0,00	1,50
**GBM 35**		**IFNγ**	13,61	41,64	0,18	0,76
**GBM 30**		**IL-2**	10,80	21,79	0,18	0,00
**GBM 31**		**IL-2**	13,08	13,51	0,00	3,36
**GBM 35**		**IL-2**	8,16	31,27	0,01	0,00
**GBM 30**		**TNFα**	74,11	60,01	0,96	3,11
**GBM 31**		**TNFα**	78,82	65,47	2,85	0,00
**GBM 35**		**TNFα**	16,75	58,78	0,00	1,44
**GBM 30**	**CD3+/ CD4- CD8-**	**IFNγ**	8,83	3,17	0,05	0,00
**GBM 31**		**IFNγ**	7,36	4,95	0,01	0,34
**GBM 35**		**IFNγ**	4,77	13,48	0,01	0,21
**GBM 30**		**IL-2**	5,17	0,52	0,09	0,00
**GBM 31**		**IL-2**	3,41	2,99	0,00	1,36
**GBM 35**		**IL-2**	1,40	22,45	0,00	0,24
**GBM 30**		**TNFα**	35,86	5,40	0,24	0,12
**GBM 31**		**TNFα**	22,96	8,07	2,35	0,25
**GBM 35**		**TNFα**	9,56	37,01	0,00	0,89

Intracellular cytokine staining: Before/after NY-ESO-1 peptide stimulation of PBMCs from patients with glioblastoma, medium values (i.e. T-cells cultured in cytokines IL-2, IL-15 and IL-21, yet without stimulating peptide targets) are already subtracted in the NY-ESO-1 antigen responses.

Supplementary Table 5: Cytokine production of NY-ESO-1 stimulated PBMCs from a Healthy Donor

T cell Subgroup	Cytokine Production	T0	TH
**CD3+CD8+**	**IFN-γ**	**0.01**	**0.18**
	**TNF-α**	**0.23**	**0.12**
	**IL-2**	**0.00**	**0.03**
**CD3+CD4+**	**IFN-γ**	**0.07**	**0.07**
	**TNF-α**	**0.03**	**0.83**
	**IL-2**	**0.00**	**0.77**
**CD3+CD4-CD8-**	**IFN-γ**	**0.00**	**0.11**
	**TNF-α**	**0.17**	**0.43**
	**IL-2**	**0.00**	**1.17**

PBMCs from HLA-A2+ healthy donors were expanded in IL-2, IL-15 and IL-21 and stimulated with the TAA NY-ESO-1 as described in the material and method section. T0= time point zero, start of the T-cell expansion; TH= time of T-cell harvest. Cytokines were measured after intracellular cytokine staining by flow cytometry. A panel of cytokines (IFN-γ, TNF-α and IL-2) were tested and the frequency of cytokine producing T-cells within the respective T-cell population (CD4, CD8 or DN) is reported at time point zero and at the time point of harvest. Numbers represent the frequency of cytokine producing T-cells in the respective CD4+, CD8+ or DN (CD4-CD8-) CD3+ T-cell population. Note that NY-ESO-1 reactive T-cells can be expanded from individuals without cancer, which reflects the fact that NY-ESO-1 reactive T-cell clones exist in the TCR repertoire in PBMCs from healthy individuals.

Supplementary Figure 1a

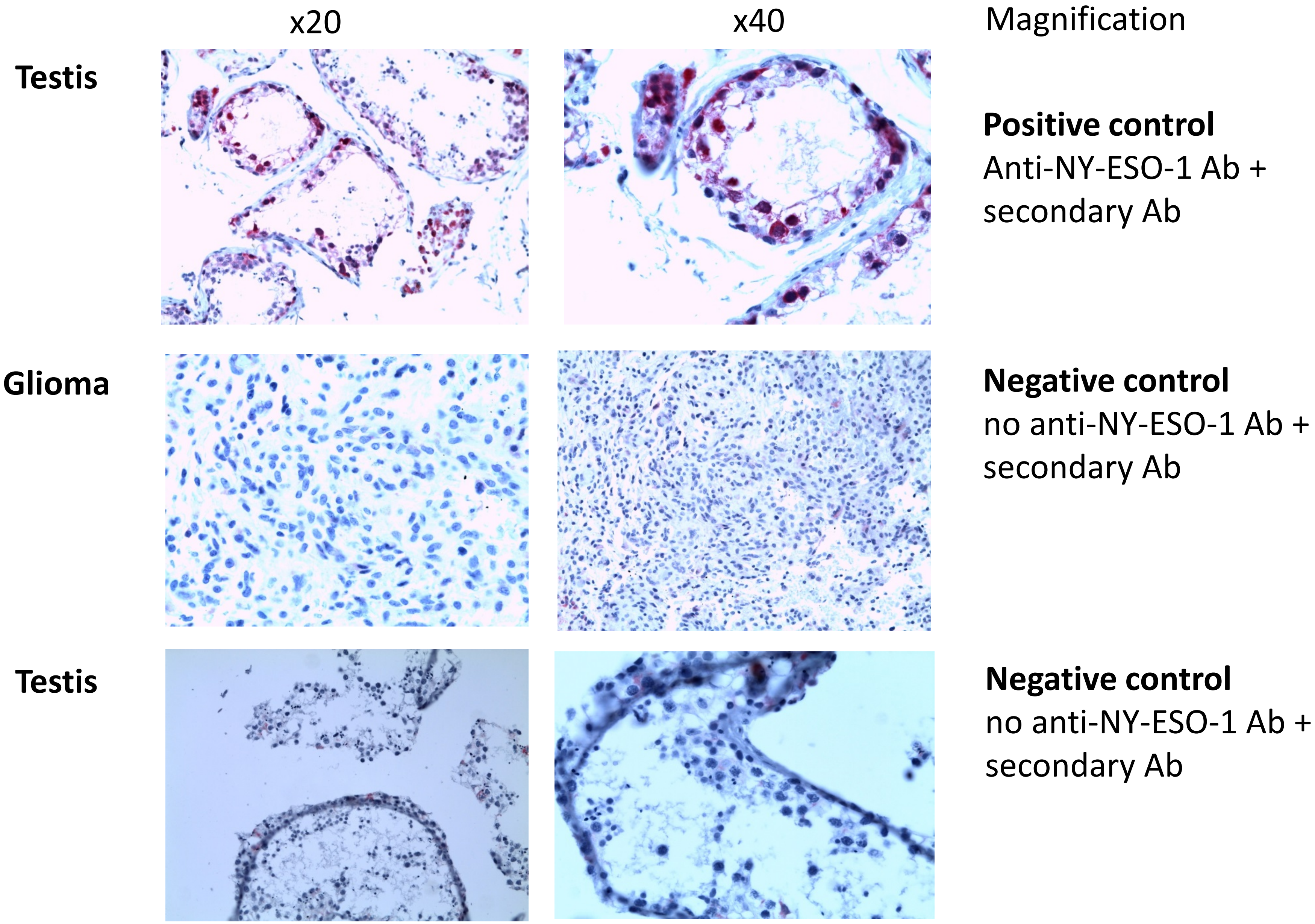

Immunohistology of NY-ESO-1. Tesis tissue stainging served as positive control. For negative controls, testis tissue as well as glioblastoma sections were stained only with the secondary antibody .

Supplementary Figure 1b

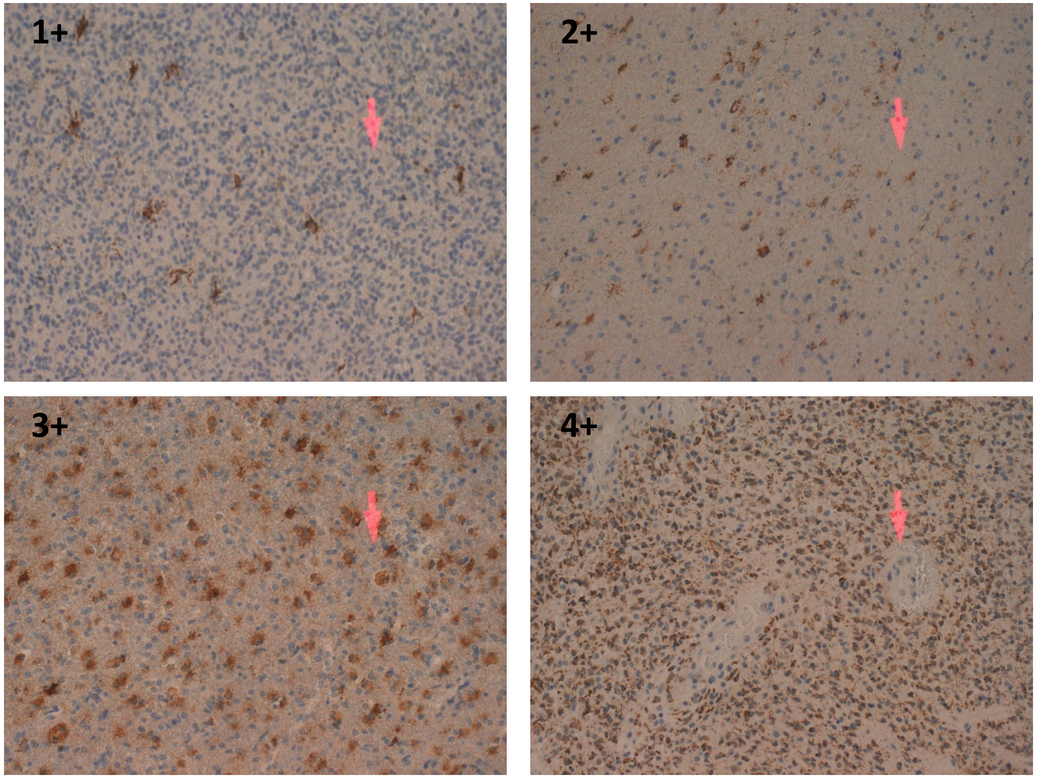

Score	Survivin positive
1+	< 10%
2+	10-20%
3+	20-50%
4+	> 50%

Immunohistology of survivin. The percentage of positive cells was evaluated using a semi-quantitative score: 1+ <10%, score 2+ = 10-20%, score 3+ = 20-50% and score 4+ >50%.

Supplementary Figure 2a

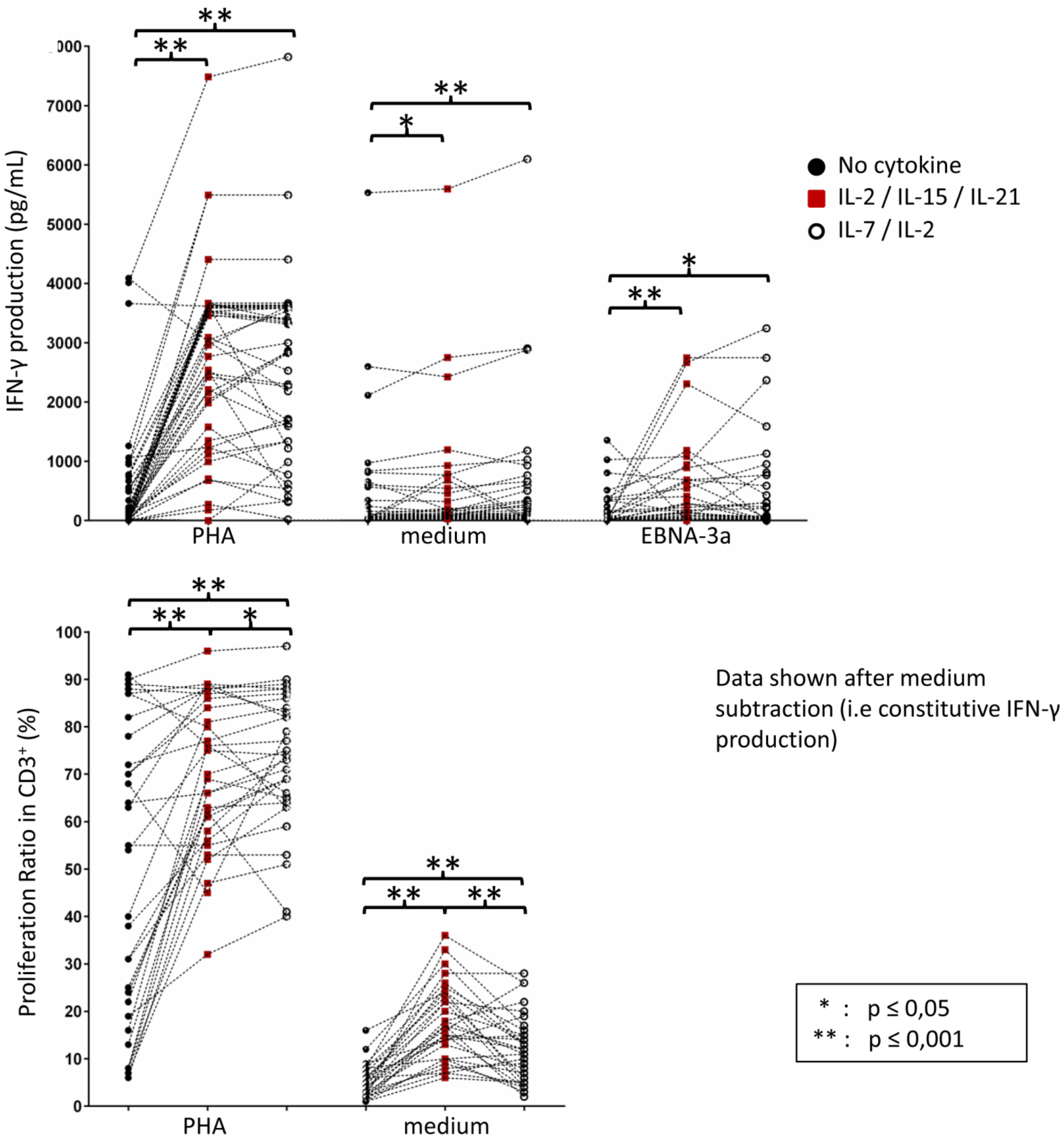

IFNγ production (top) and proliferation ratio (bottom) after 7 day expansion of patients' peripheral blood lymphocytes with positive controls (PHA, or the viral target EBNA-3) and a negative control (medium only); 3 different culture conditions: i) without cytokines (RPMI only), ii) with a IL-7/IL-2 cytokine cocktail, or iii) with a IL-2/IL-15/IL-21 cytokine cocktail (* p ≤ 0,05, ** p ≤ 0,001).
Data shown after subtraction of the constitutive IFNγ production.

Supplementary Figure 2b

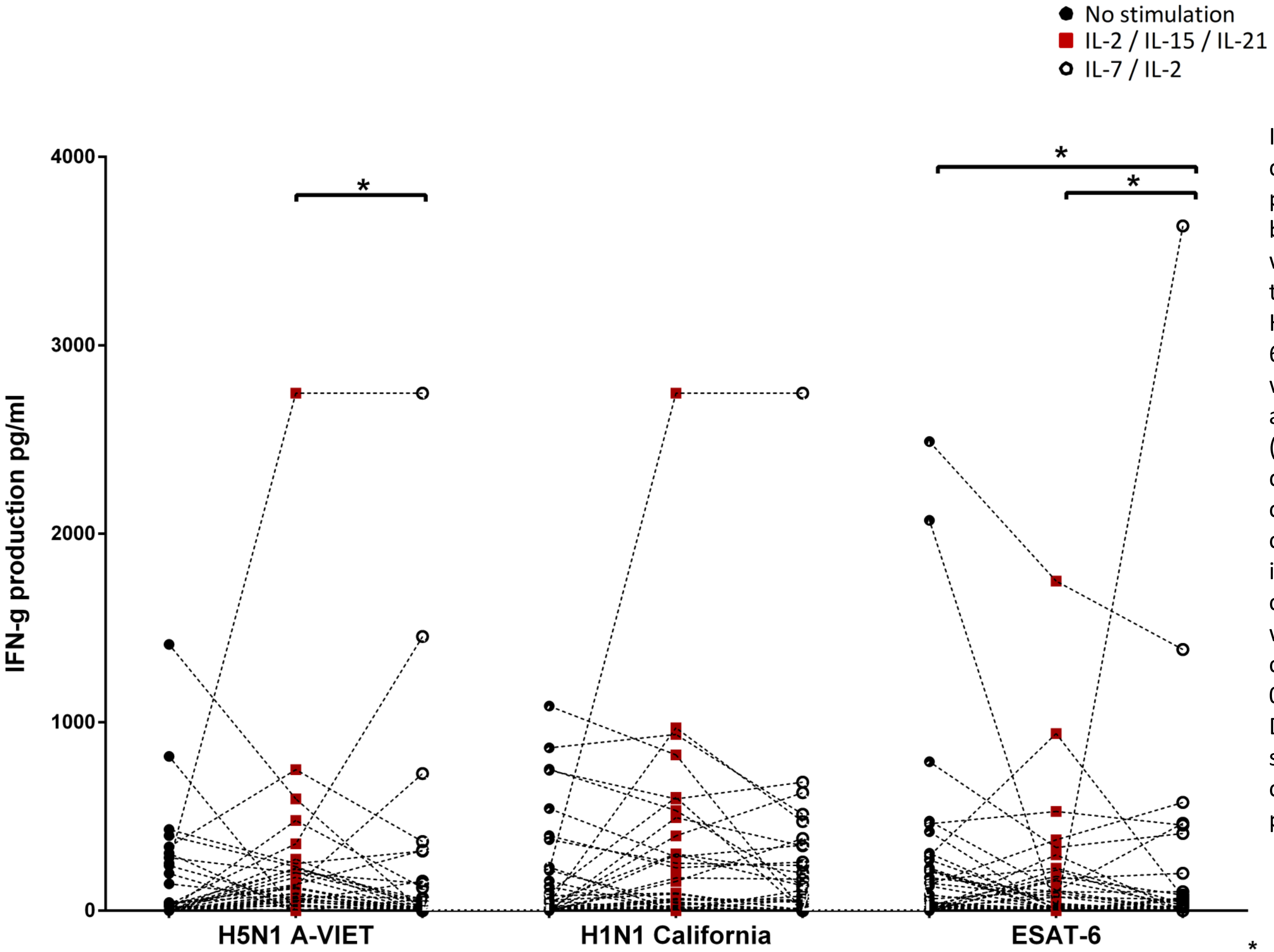

IFNγ production after 7 day expansion of patients' peripheral blood lymphocytes with controls (the viral target H1N1 California, H5N1 A-VIET or ESAT-6, antigen associated with M. tuberculosis) and a negative control (medium only); 3 different culture conditions: i) without cytokines (RPMI only), ii) with a IL-7/IL-2 cytokine cocktail, or iii) with a IL-2/IL-15/IL-21 cytokine cocktail (* p ≤ 0,05, ** p ≤ 0,001). Data shown after subtraction of the constitutive IFNγ production.

* P<0.05
** P<0.001

Supplementary Figure 3

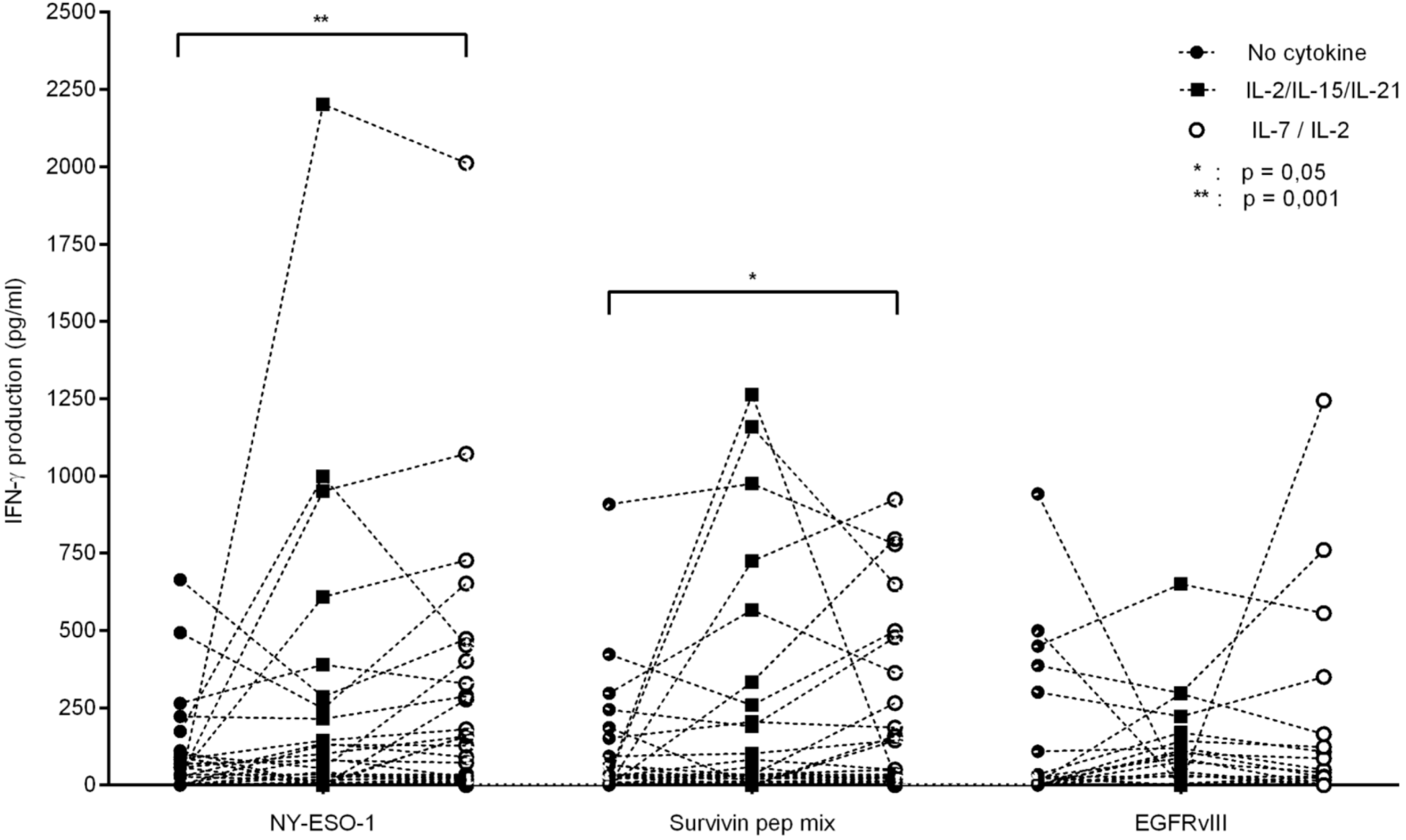

IFNγ production after a 7 day expansion of peripheral blood with NY-ESO-1, the survivin peptide mix or the EGFRvIII antigen; 3 different conditions: i) without cytokines (RPMI only), ii) with a IL-7/IL-2 cytokine cocktail, or iii) with a IL-2/IL-15/IL-21 cytokine cocktail (* p ≤ 0,05, ** p ≤ 0,001). Data shown after subtraction of the constitutive IFNγ production.

Supplementary Figure 4

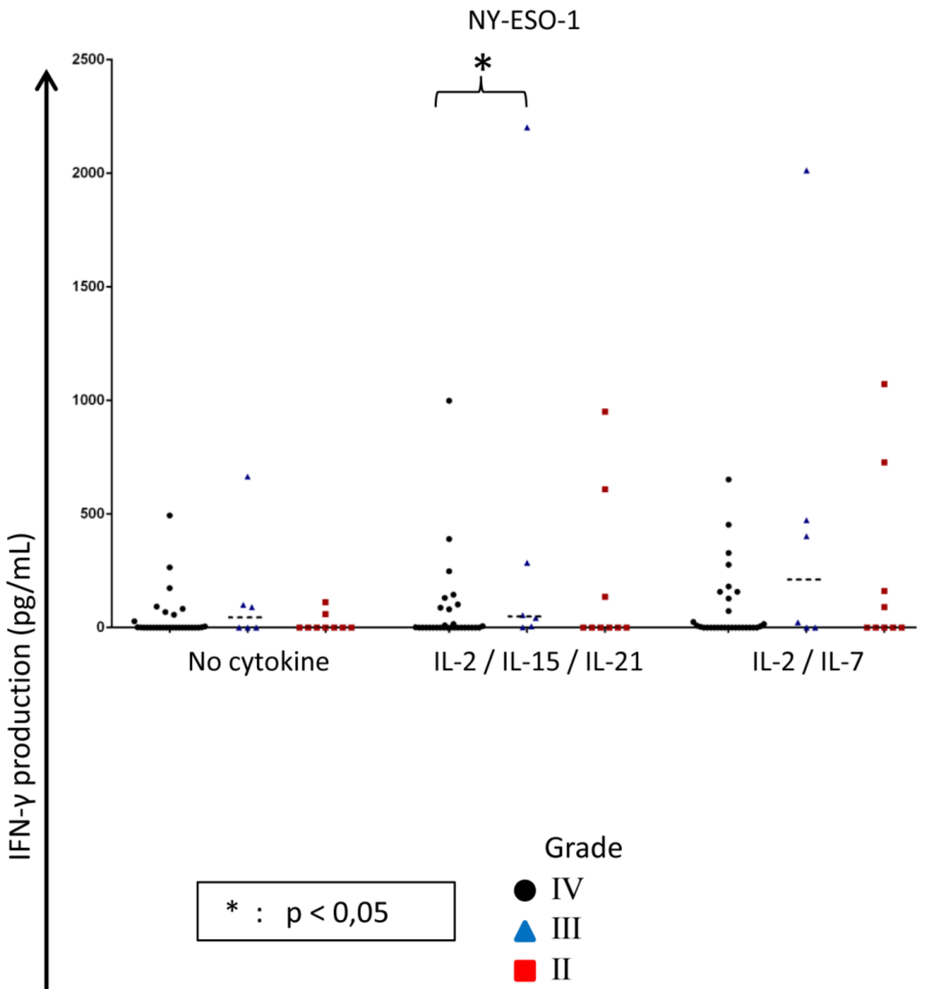

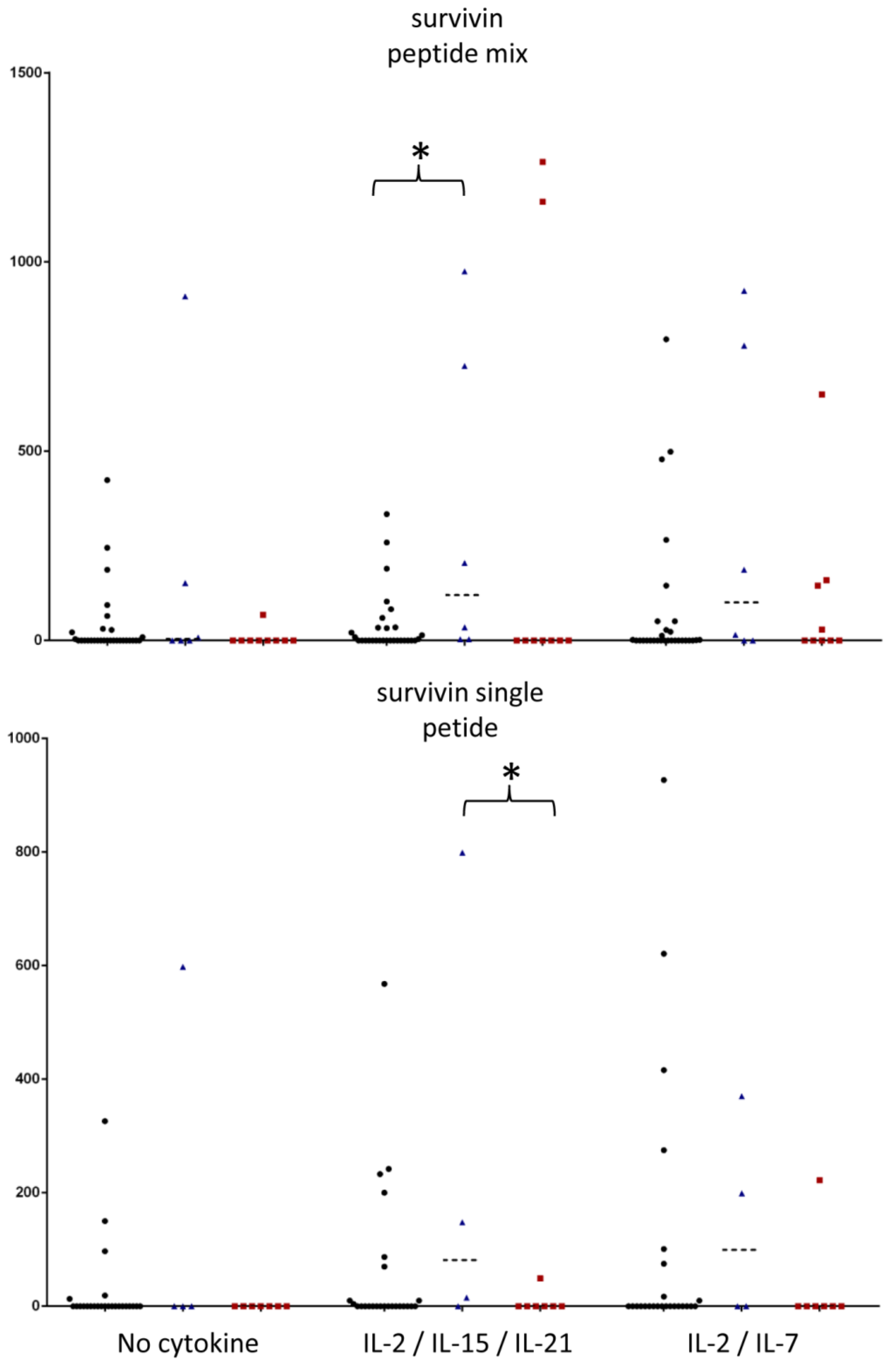

IFNγ production (pg/mL) comparing the different histopathological glioma grades (II, III, IV) after a 7 day expansion of peripheral blood with peptide antigens (NY-ESO-1, survivin peptide mix and survivin single peptide) using 3 different conditions: i) without cytokine (RPMI only), ii) with a IL-7/IL-2 cytokine cocktail, or iii) with a IL-2/IL-15/IL-21 cytokine cocktail. Horizontal dotted lines show the median values. (* p ≤ 0,05). Data shown after subtraction of the constitutive IFNγ production.

Supplementary Figure 5

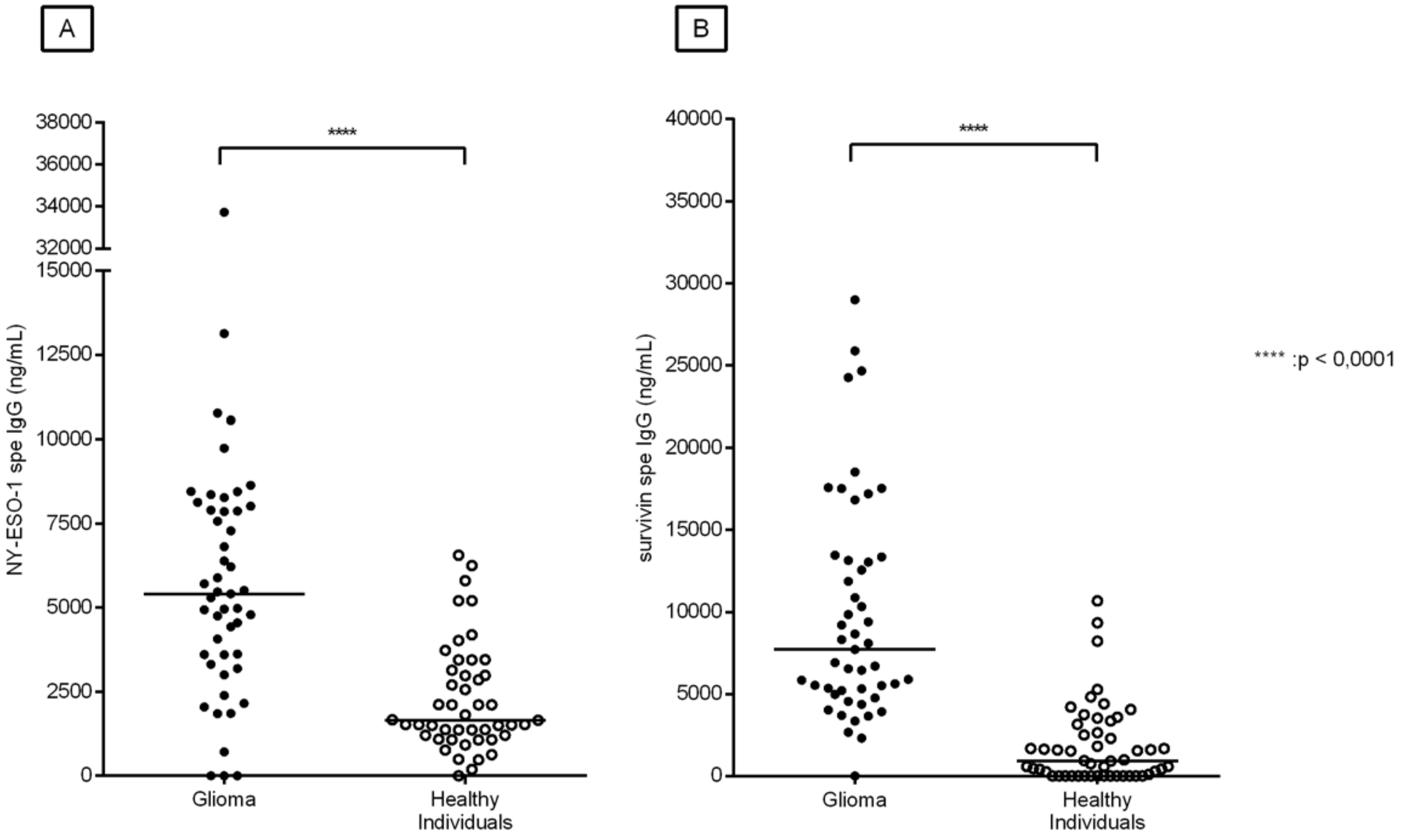

Anti-NY-ESO-1 (A) and anti-survivin (B) plasma humoral immune response from patients with glioma and age and gender-matched healthy individuals (**** p < 0,0001). Medians are indicated. Strong IgG responses in plasma from patients with glioma directed against TAAs.

Supplementary Figure 6

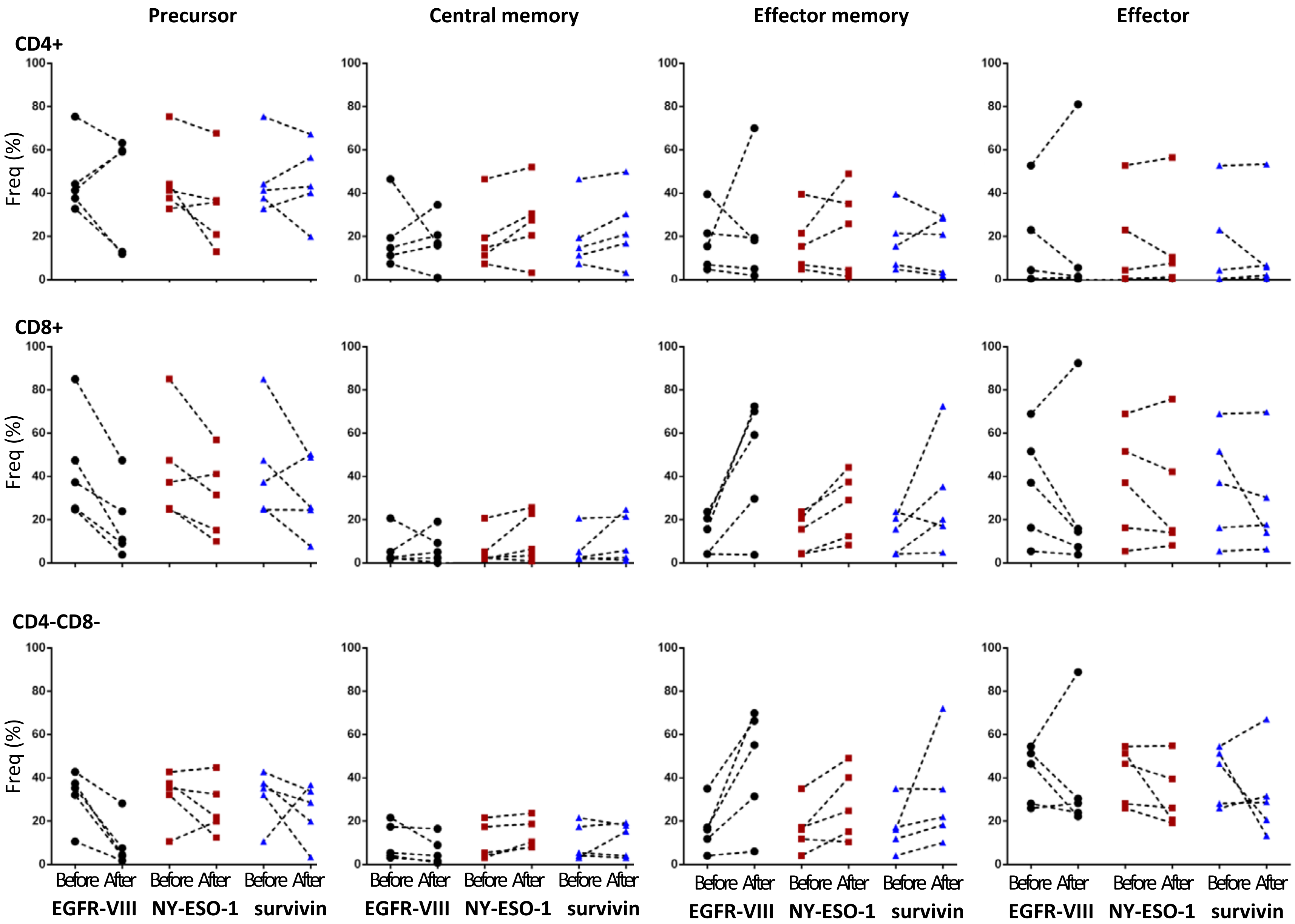

PBMCs from patients with GBM were expanded using the IL-2, IL-15 and IL-21 cytokine cocktail and NY-ESO-1 peptides as described in the material and methods section. Before: time point prior to culture initiation; After = Time of harvest, i.e. day 18. PBMCs gated on CD3+ T-cells, then on CD4+ or CD8+ T-cells and analyzed for CD45RA and CCR7 marker expression. Note the frequency of precursor CD45RA+CCR7+ T-cells. Numbers represent frequency of T-cells in the respective parental population.

Supplementary Figure 7

4-1BB expression increased after expansion with NY-ESO-1

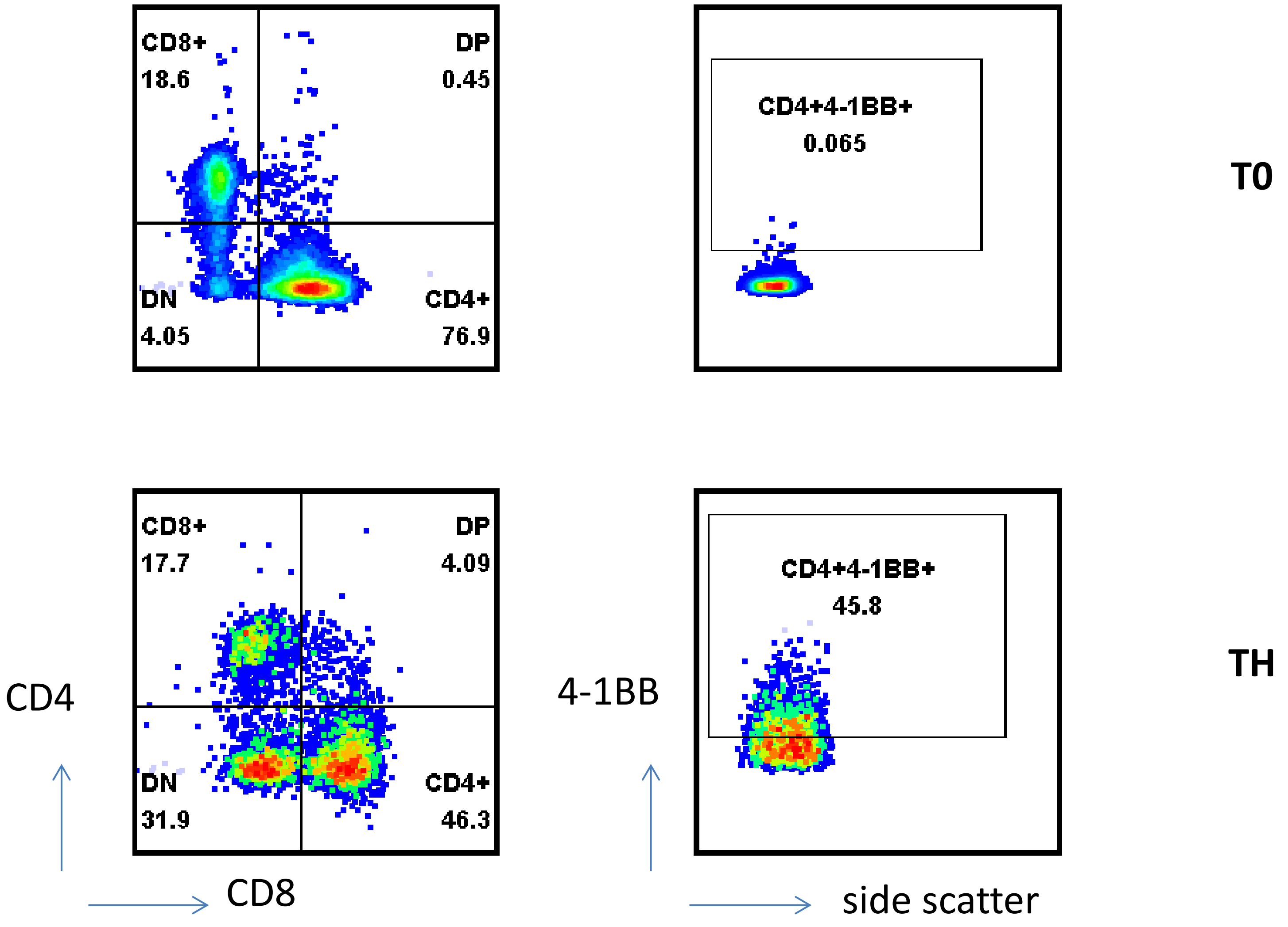

30 million cells from a patient with GBM were expanded using the IL-2, IL-15 and IL-21 cytokine cocktail and NY-ESO-1 peptides as described in the material and methods section. T0= time point prior to culture initiation; TH= Time of harvest, i.e. day 18. Top panel: PBMCs gated on CD3+CD4+ T-cells analyzed for 4-1BB expression. Bottom panel: T-cells were gated on CD3+ 4+ T-cells and the frequency of 4-1BB+ T-cells were determined. Numbers represent frequency of T-cells in the respective parental population. Frequency of 4-1BB + T-cells in CD3+CD4+ T-cells; side scatter versus 4-1BB marker expression.

Supplementary Figure 8

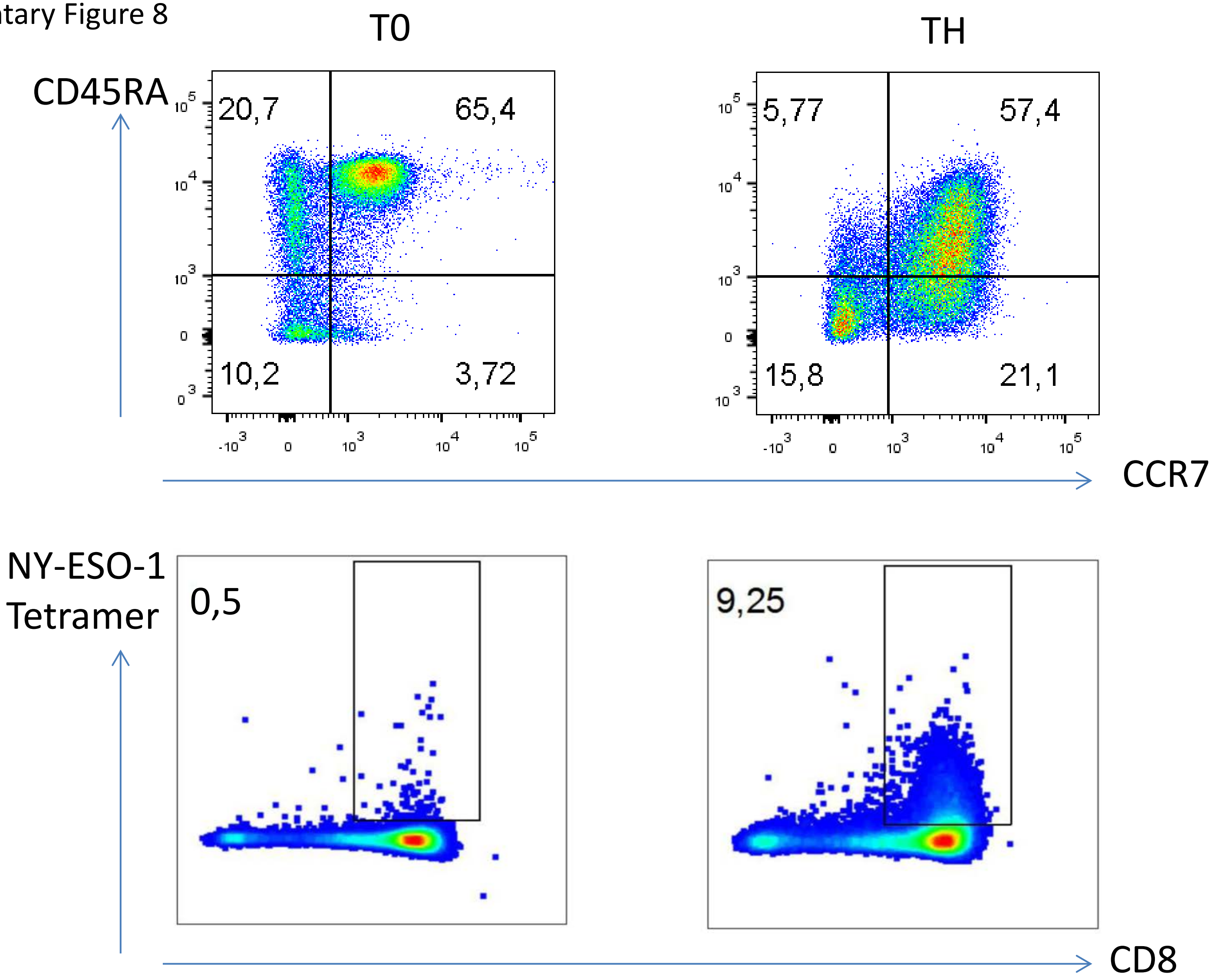

30 million cells from an HLA-A*02:01+ patients with GBM were expanded using the IL-2, IL-15 and IL-21 cytokine cocktail and NY-ESO-1 peptides as described in the material and methods section. T0= time point prior to culture initiation; TH= Time of harvest, i.e. day 18. Top panel: PBMCs gated on CD3+CD8+ T-cells analyzed for CD45RA and CCR7 marker expression. Note the frequency of precursor CD45RA+CCR7+ T-cells. Bottom panel: T-cells were gated on CD3+ T-cells and tetramer – reative T-cells were tested at day 18. Left: negative tetramer, right: NY-ESO-1 tetramer; 1.25 NY-ESO-1 tetramer – positive T-cells at time point zero (data not shown). Numbers represent frequency of T-cells in the respective parental population.

Supplementary Figure 9

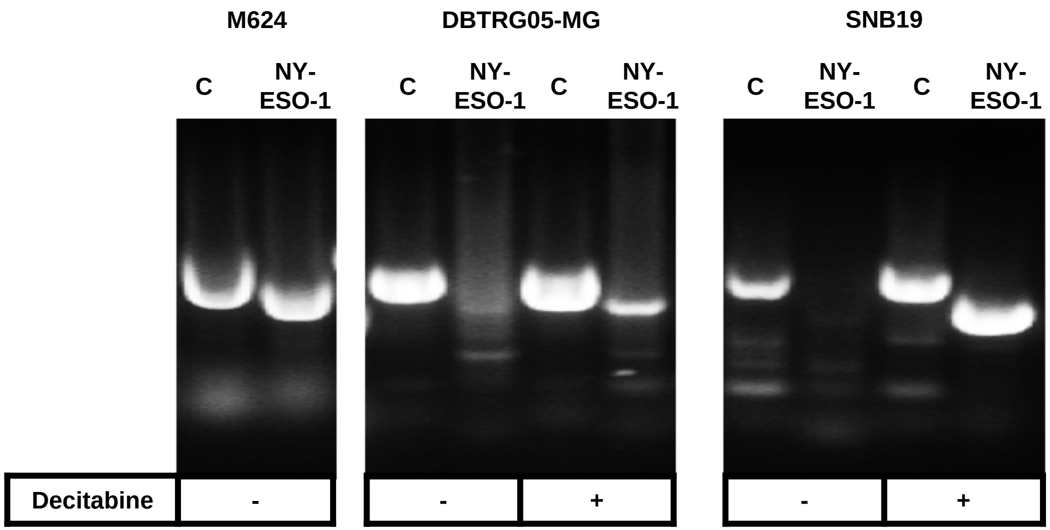

pg/ml	M624		DBTRG05MG				SNB19			
**Decitabine**	-		-		+		-		+	
**T-cells from:**	**Tumor**	**W6/32**	**Tumor**	**W6/32**	**Tumor**	**W6/32**	**Tumor**	**W6/32**	**Tumor**	**W6/32**
**dextramer sorted**	63,52	12,90	63,81	25,17	94,66	23,34	60,42	10,69	79,82	22,04
**IFNγ capture**	1197,82	119,43	791,13	384,88	1254,23	372,04	186,89	18,13	756,86	285,85

NY-ESO-1 expression in 3 different tumor cell lines: melanoma M624 and glioblastoma DTRG05-MG and SNB19 upon decitabine treatment (-/+). GADPH served as the positive control (C) . IFNγ production (%) from isolated NY-ESO-1 T-cells from two glioblastoma patients using dextramer sorting or a IFNγ capture procedure after 3 days co-culture with the tumor cell lines M624, DTRG05-MG and SNB19 treated with (+) or without (-) decitabine. Tumor cells without T-cells or T-cells without tumor cells served as controls (data not shown). Anti-tumor reactivity could be blocked with the anti-MHC class I directed mAb W6/32. Numbers are pg/mL. Left columns represent cytokine production (tumor+ T-cells); right columns: anti-MHC class I blocking of T-cell anti-tumor reactivity.
